# Structure and function of a glycoside hydrolase family 8 endoxylanase from *Teredinibacter turnerae*

**DOI:** 10.1107/S2059798318009737

**Published:** 2018-10-02

**Authors:** Claire A. Fowler, Glyn R. Hemsworth, Fiona Cuskin, Sam Hart, Johan Turkenburg, Harry J. Gilbert, Paul H. Walton, Gideon J. Davies

**Affiliations:** a York Structural Biology Laboratory, Department of Chemistry, The University of York, York YO10 5DD, England; b School of Molecular and Cellular Biology, The Faculty of Biological Sciences, University of Leeds, Leeds LS2 9JT, England; c School of Natural and Environmental Science, Newcastle University, Newcastle upon Tyne NE1 7RU, England; d Institute for Cell and Molecular Biosciences, Newcastle University, Newcastle upon Tyne NE2 4HH, England; e Department of Chemistry, The University of York, York YO10 5DD, England

**Keywords:** glycoside hydrolase, biomass, biofuels, marine polysaccharides, cellulolytic enzymes, shipworms, *Teredinibacter turnerae*

## Abstract

The biological conversion of lignocellulosic matter into high-value chemicals or biofuels is of increasing industrial importance as the sector slowly transitions away from nonrenewable sources. Many industrial processes involve the use of cellulolytic enzyme cocktails – a selection of glycoside hydrolases and, increasingly, polysaccharide oxygenases – to break down recalcitrant plant polysaccharides. ORFs from the genome of *Teredinibacter turnerae*, a symbiont hosted within the gills of marine shipworms, were identified in order to search for enzymes with desirable traits. Here, a putative *T. turnerae* glycoside hydrolase from family 8, hereafter referred to as TtGH8, is analysed. The enzyme is shown to be active against β-1,4-xylan and mixed-linkage (β-1,3,β-1,4) marine xylan. Kinetic parameters, obtained using high-performance anion-exchange chromatography with pulsed amperometric detection and 3,5-dinitrosalicyclic acid reducing-sugar assays, show that TtGH8 catalyses the hydrolysis of β-1,4-xylohexaose with a *k*_cat_/*K*_m_ of 7.5 × 10^7^ *M* ^−1^ min^−1^ but displays maximal activity against mixed-linkage polymeric xylans, hinting at a primary role in the degradation of marine polysaccharides. The three-dimensional structure of TtGH8 was solved in uncomplexed and xylobiose-, xylotriose- and xylohexaose-bound forms at approximately 1.5 Å resolution; the latter was consistent with the greater *k*_cat_/*K*_m_ for hexasaccharide substrates. A ^2,5^ *B* boat conformation observed in the −1 position of bound xylotriose is consistent with the proposed conformational itinerary for this class of enzyme. This work shows TtGH8 to be effective at the degradation of xylan-based substrates, notably marine xylan, further exemplifying the potential of *T. turnerae* for effective and diverse biomass degradation.

## Abstract 2

The symbionts of marine shipworms provide a rich reservoir of potential carbohydrate-active enzymes. Here, the 1.5 Å resolution three-dimensional structure of a *T. turnerae* GH8 xylanase is revealed and its potential in biomass degradation is highlighted.

## 1. Introduction   

**Figure 1:**
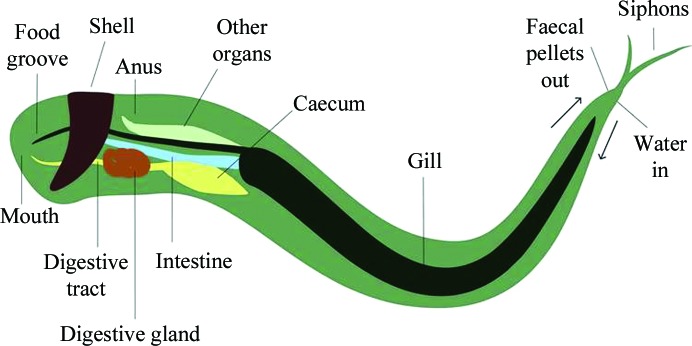
Basic diagram of a shipworm (image not to scale) showing the general layout of the main organs within the animal. More information on shipworm physiology can be found in Betcher (2011 ▸), Gallager *et al.* (1981 ▸) and Waterbury *et al.* (1983 ▸), and in-depth illustrations and descriptions can be found in Turner (1966 ▸).

The demand for biofuels is increasing amid positive shifts in political and public opinion regarding the growing need for more sustainable fuel sources (discussed, for example, in Somerville, 2007 ▸; Pauly & Keegstra, 2008 ▸). A barrier towards the sustainable and efficient usage of plant biomass for fuel conversion lies in the complexity and recalcitrance of plant cell walls (Himmel *et al.*, 2007 ▸; Bomble *et al.*, 2017 ▸). The heterogeneous matrix of various carbohydrate compounds hinders the enzymatic breakdown of plant cell walls into energy-rich carbohydrate monomers. Crystalline cellulose regions are interspersed with a web of more soluble polysaccharides, known as hemicelluloses. The plant cell-wall matrix is strengthened by a hydrophobic and insoluble barrier: a mixture of phenolic compounds known as lignin (Li *et al.*, 2015 ▸). In nature, the breakdown of lignocellulosic material is achieved through the synergistic action of a wide variety of different enzymes, including glycoside hydrolases and polysaccharide oxygenases (Hemsworth *et al.*, 2015 ▸; Walton & Davies, 2016 ▸). A single organism can produce a consortium of enzymes capable of lignocellulosic degradation or can utilize the symbiotic behaviour of other smaller organisms such as bacteria and fungi (Cragg *et al.*, 2015 ▸). One organism of increasing interest is the marine symbiont *Teredinibacter turnerae*, which has been found in the gills (Fig. 1 ▸) of at least 24 species of bivalve molluscs (Ekborg *et al.*, 2007 ▸; Horak & Montoya, 2014 ▸).

Marine bivalve molluscs of the family Teredinidae, more commonly referred to as shipworms (Fig. 1 ▸), are considered pests by the maritime community as they inflict damage onto wooden structures such as piers and ship hulls by burrowing into the material (Cragg *et al.*, 2015 ▸). In 1927, Boynton and Miller observed the disappearance of 80% of cellulose and 15–56% of hemicellulose from Douglas fir piling during its transit through the digestive tract of *Teredo navalis* (Boynton & Miller, 1927 ▸), highlighting the ability of these organisms to degrade recalcitrant plant biomass. Much later, in 1983, Waterbury and coworkers observed, isolated and cultured the symbiotic bacterium *T. turnerae* (Waterbury *et al.*, 1983 ▸) from specialized cells called bacteriocytes found inside an internal region of the shipworm’s gills. In its genome, *T. turnerae* encodes >100 glycoside hydrolases, enzymes that are capable of breaking glycosidic bonds, 54% of which have been classed as ‘wood-specific’ (Yang *et al.*, 2009 ▸). Furthermore, *T. turnerae* enzymes have been identified in the shipworm gut (O’Connor *et al.*, 2014 ▸), thus establishing the resident bacteria and the enzymes that they produce as key players in shipworm biology. It is therefore now clear that lignocellulose degradation may occur through the synergistic action of host enzymes and enzymes from this community of endosymbiotic bacteria housed within the gills of the shipworm.

In this light, we sought to mine the uncharacterized *T. turnerae* glycoside hydrolases to find enzymes that would be active under more biotechnologically relevant conditions and on useful substrates. *T. turnerae* exists primarily in high-salt conditions, and so its enzymes may be additionally stabilized towards harsher environments such as might be experienced in a biorefinery. As a proof of principle, we chose to focus on the consortia of enzymes harnessed by this organism to degrade xylans, given the interest in this substrate in the context of biofuels (Somerville, 2007 ▸; Pauly & Keegstra, 2008 ▸; Biely *et al.*, 2016 ▸), and also in the pulp and paper, animal feed and bread-making sectors. Additionally, following the recent insights gained from studying xylan-degrading enzyme consortia from new ecological niches (Rogowski *et al.*, 2015 ▸), work that revealed both new enzymes and specificities, the study of xylan-degrading enzymes from this shipworm symbiont represents fertile new ground for further discoveries in this area.

Applying the CAZY classification for carbohydrate-active enzymes (http://www.cazy.org; see Lombard *et al.*, 2014 ▸) to the *T. turnerae* genome reveals many potential xylanases, including ten GH10 enzymes, five GH11 enzymes and two enzymes from the potential xylanase-containing family GH30 (all of these glycoside hydrolase families are reviewed in CAZypedia; for a review, see The CAZypedia Consortium, 2018 ▸). We were particularly drawn to family GH8, a cellulase/xylanase-containing family, from which *T. turnerae* has just a single representative, TtGH8. We sought to use this enzyme as an exemplar for whether *T. turnerae* can provide enzymes with biological and chemical utility, cast in terms of three-dimensional structure and notably, in this case, in the context of a diverse array of other potential xylanolytic enzymes.

Here, we report the cloning, expression and characterization of this GH8 carbohydrate-active enzyme (Lombard *et al.*, 2014 ▸). We show that TtGH8 is a xylan-active endoxylanase with six catalytically relevant subsites and notably a maximal activity towards mixed-linkage (β-1,3,β-1,4) marine xylan. The three-dimensional structure of TtGH8, determined to 1.5 Å resolution, reveals intimate details of the substrate-binding sites and the distortions of xylose within the catalytic centre. The work thus highlights the potential of the *T. turnerae* genome for enzyme discovery and adds to the growing toolbox of enzymes that may be used to tackle the recalcitrant hemicellulose xylan (Biely *et al.*, 2016 ▸).

## 2. Materials and methods   

### 2.1. Purification of TtGH8 and catalytic mutants   

The sequence of TtGH8 (TERTU_4506; UniProt C5BJ89) was analysed, and the catalytic domain was identified (Supplementary Table S1) and truncated to remove the original signal peptide and linker region and to include an N-terminal hexahistadine tag and 3C protease cleavage site. The sequence was codon-optimized and synthesized for expression in *Escherichia coli* by GenScript (New Jersey, USA) and the plasmid was transformed into *E. coli* BL21 competent cells (Supplementary Table S2). Catalytic residues were identified using the literature and structures of similar GH8 proteins deposited in the PDB. The mutations were designed using custom primers and implemented using the Q5 site-directed mutagenesis kit (NEB) to alter Asp281 to Asn (TtGH8 D281N; Supplementary Table S3). Expression testing for both constructs was carried out prior to large-scale production. Cultures (6 × 500 ml LB, 30 mg ml^−1^ kanamycin) were inoculated with 500 µl overnight culture and grown at 37°C and 200 rev min^−1^ until an OD of 0.6 was obtained. IPTG (1 m*M* final concentration) was then added and the cultures were cooled to 16°C and left shaking overnight. The cultures were harvested by centrifugation and the pellet was resuspended in 50 m*M* HEPES, 250 m*M* NaCl, 30 m*M* imidazole pH 7. The cells were lysed by sonication and centrifuged at 15*g* for 30 min. The supernatant was collected and loaded onto a pre-equilibrated Nickel HiTrap Crude 5 ml affinity column. Nickel-affinity chromatography was run on an ÄKTA start, with an elution gradient of 30–300 m*M* imidazole over 25 column volumes. The collected protein sample was treated with a 1:100 ratio of 3C protease to TtGH8 and 5 m*M* DTT and left shaking at room temperature overnight. The sample was loaded onto a pre-equilibrated Nickel HiTrap Crude 5 ml affinity column and the flowthrough and wash were collected. The collected sample was buffer-exchanged into 20 m*M* HEPES, 200 m*M* NaCl before being concentrated to approximately 300 µl and loaded onto a Superdex 200 gel-filtration column. Pure fractions were combined, concentrated and buffer-exchanged into 10 m*M* HEPES. To check their purity, samples were analysed by SDS–PAGE throughout purification and the final sample was analysed by electrospray ionization mass spectrometry.

### 2.2. Thermal shift analysis (TSA)   

Samples (30 µl total) containing SYPRO Orange dye (15 µl) and enzyme (final concentration of 1 mg ml^−1^) with either buffer or substrate were prepared and thermal shift analyses were run on a Stratagene Mx3005P qPCR device. The substrates tested included the solid substrates (small amount, unmeasured) tamarind xyloglucan, rye arabinoxylan, birchwood xylan, shrimp shell chitin and Avicel, and the soluble oligosaccharides cellohexaose, cellobiose, xylobiose and xylohexaose (10 m*M*). Samples were heated from 20 to 91°C in increments of 1°C over 71 cycles. The fluorescence of SYPRO Orange was monitored throughout and the data were used to calculate the melting temperature of the protein (Supplementary Fig. S1). The data were analysed using the *JTSA* fitting program developed by Paul Bond, which is available at http://paulsbond.co.uk/jtsa/#/input.

### 2.3. Thin-layer chromatography (TLC) and liquid chromatography–mass spectrometry (LCMS)   

**Figure 2:**
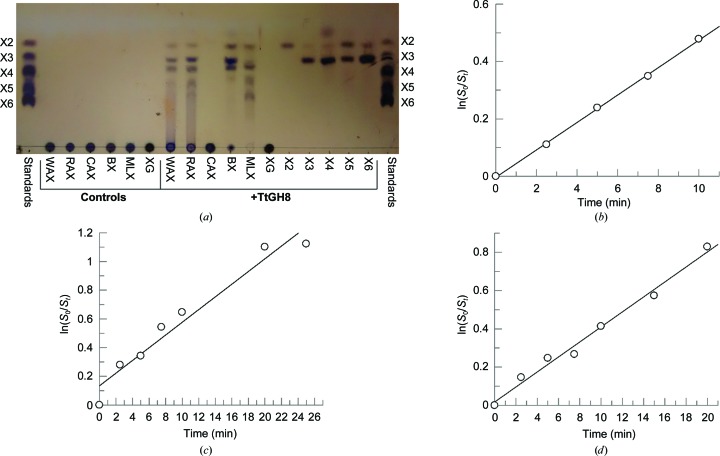
(*a*) TtGH8 activity was explored on a range of substrates using thin-layer chromatography. Hydrolysis activity can be observed on WAX, RAX, BX and MLX, as well as on the oligosaccharides xylotetraose (X4), xylopentaose (X5) and xylohexaose (X6). Activity analysis of TtGH8 on xylohexaose (*b*), xylopentaose (*c*) and xylotetraose (*d*) was carried out with HPAEC-PAD by substrate depletion. The peak areas observed in the HPAEC-PAD traces were normalized against a fucose internal standard and values for ln(*S*_0_/*S*_*t*_), where *S*_0_ is the substrate-peak area at time 0 and *S*_*t*_ is the substrate-peak area at time *t*, were plotted against time. Linear regression analysis was used to calculate *k*_cat_/*K*_m_ (in *M* ^−1^ min^−1^).

Xylo-oligosaccharides were purchased commercially: xylobiose (X2) from Sigma and TCP, and xylotriose (X3), xylotetraose (X4), xylopentaose (X5), xylohexaose (X6) and polysaccharides from Megazyme (unless stated otherwise). Overnight hydrolysis reactions with the xylo-oligosaccharides X2–X6 (1 m*M*), wheat arabinoxylan (WAX), rye arabinoxylan (RAX), corn arabinoxylan (CAX), birchwood xylan (BX), mixed-linkage β1–3,β1–4 xylan (MLX, purchased from Elicityl/Oligotech) and xyloglucan (XG) at 1 mg ml^−1^ were incubated at 37°C with 1 µ*M* TtGH8. The samples were heated at 90°C prior to spotting onto a TLC plate (total 4 µl). Standards containing X2–X6 at 1 m*M* each were run on the same plate. TLC plates were placed in chromatography tanks containing the running buffer [50%(*v*/*v*) *n*-butanol, 25%(*v*/*v*) acetic acid, 25%(*v*/*v*) water]. Plates were run once, dried and then re-run to improve the separation of sugars. The plates were visualized using a staining solution [3%(*v*/*v*) sulfuric acid, 75%(*v*/*v*) ethanol, 0.1%(*w*/*v*) orcinol monohydrate], dried and then heated to approximately 100°C (Fig. 2 ▸ *a*). Hydrolysis samples for LCMS were prepared using 50 m*M* ammonium acetate buffer pH 6, approximately 1 mg ml^−1^ substrate and 1 µ*M* enzyme. Samples were incubated at 37°C overnight and shaken at 500 rev min^−1^. If required, samples were centrifuged to remove any solid materials and 100 µl was loaded onto a Cosmosil Sugar-D HPLC column using the LC-MS Dionex system, where the separated products were analysed by ESI or PAD mass spectrometry. Running buffers were a mixture of water and acetronitrile, with some test runs also including 1% formic acid (Supplementary Figs. S2 and S3).

### 2.4. Kinetics measurements using high-performance anion-exchange chromatography with pulsed amperometric detection (HPAEC-PAD)   

Substrate-depletion kinetics measurements were performed on TtGH8 with xylohexaose, xylopentaose and xylotetraose and were measured using an HPAEC-PAD Dionex system. Hydrolysis reactions were run at 37°C using different substrate and enzyme concentrations, with aliquots removed at set time points and boiled to inactivate TtGH8. All samples were mixed with a fucose internal standard and run on an anion-exchange column (CarboPac) using a sodium acetate gradient. The depletion of substrate during the reaction can be related to *k*_cat_/*K*_m_ through 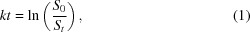
where *k* = (*k*_cat_/*K*_m_)[enzyme], *t* is time and *S*_0_ and *S*_*t*_ are the substrate-peak areas at time 0 and *t*, respectively. The substrate-peak areas observed in the HPAEC PAD traces were normalized against both an external and internal fucose standard and the resulting values for ln(*S*_0_/*S*_*t*_) were plotted against time, producing positive gradients (the change in the substrate-peak area increases from 0, as the substrate-peak area at *t* = 0 is at its maximum). Linear regression analysis was used to measure the gradient that represents the rate of reaction, and *k*_cat_/*K*_m_ (*M* ^−1^ min^−1^) was determined by dividing this gradient by the enzyme concentration (Figs. 2 ▸ *b*, 2 ▸ *c* and 2 ▸ *d*). The use of substrate depletion to obtain *k*_cat_/*K*_m_ has been widely used in the glycoside hydrolase field (see, for example, Pell *et al.*, 2004 ▸; Charnock *et al.*, 1998 ▸; Matsui *et al.*, 1991 ▸).

### 2.5. Reducing-sugar assay   

**Figure 3:**
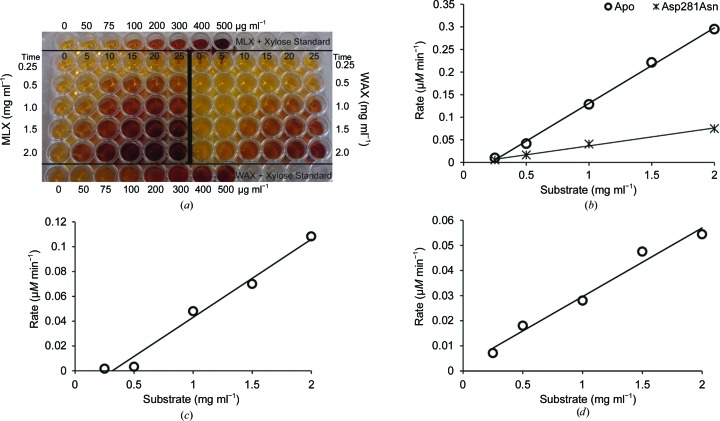
TtGH8 activity on polysaccharides was determined using the 3,5-dinitrosalicyclic acid (DNSA) reducing-sugar assay. (*a*) Photograph of the plate containing aliquots representing different reaction time points (0–25 min) and substrate concentrations [mixed-linkage xylan (MLX) and wheat arabinoxylan (WAX) at 0.5–2.0 mg ml^−1^]. Reaction of the DNSA agent with reducing sugars causes a visible colour change from light yellow to deep brown, with the amount of colour change measured by the absorbance at 510 nm being equivalent to the amount of reducing sugar (chain ends) produced during the enzyme reaction. Standard curves were calculated using the free xylose controls in the top and bottom rows of the plate. These were MLX (1 mg ml^−1^) + xylose (0–500 µg ml^−1^) and WAX (1 mg ml^−1^) + xylose (0–500 µg ml^−1^). TtGH8 is more active on MLX than on WAX as there is a greater colour change. Absorption measurements taken for each reaction aliquot can be converted into the amount of reducing sugar present using the standard curve [shown in (*a*)] and plotted against time to give a rate (µ*M* min^−1^, not shown). The rates for each reaction condition are plotted against the substrate concentration, where the gradient divided by the enzyme concentration is equivalent to *k*_cat_/*K*_m_. (*b*) Activity of TtGH8 and TtGH8 catalytic mutant on MLX, (*c*) activity of TtGH8 on WAX and (*d*) activity of TtGH8 on BX.

The activity of TtGH8 towards several polysaccharides was tested using the 3,5-dinitrosalicyclic acid (DNSA) reducing-sugar assay. Hydrolysis reactions with enzyme and substrate were run at 37°C and 150 µl aliquots were removed and immediately mixed in a 1:1 volume ratio with the DNSA agent [comprising 1%(*w*/*v*) DNSA, 0.2%(*v*/*v*) phenol, 1%(*w*/*v*) NaOH, 0.002% glucose and 0.05%(*w*/*v*) Na_2_SO_3_] at set time points to stop the reaction. The aliquots were heated at 90°C for 20 min and cooled on ice for 10 min. The absorbance of each sample was measured at 575 nm. A standard curve of 0–500 µg ml^−1^ xylose (plus 1 mg ml^−1^ polysaccharide substrate) was used to quantify the released reducing sugar. Reaction rates determined for each different substrate-concentration condition were plotted against the substrate concentration, where the gradient divided by the enzyme concentration is *k*_cat_/*K*_m_ (Fig. 3 ▸).

### 2.6. Crystallization and X-ray crystallography of TtGH8 and mutants   

Initial crystallization screening was performed robotically using a Mosquito crystal robot and commercial screens, including Crystal Screen HT and Index from Hampton Research and PACT from Molecular Dimensions. Several crystal hits were obtained for TtGH8 and TtGH8 D281N. A 24-well optimization screen containing 0.1 *M* sodium acetate pH 4.6–5.2, 0.2 *M* NaCl, 14–24% polyethylene glycol (PEG) 6000 was used to produce the final crystallization condition for TtGH8: 0.1 *M* sodium acetate pH 5.0, 0.2 *M* NaCl, 16% polyethylene glycol. Thin rod-shaped crystals were harvested in nylon loops and cryoprotected by soaking them in mother liquor plus 30%(*v*/*v*) ethylene glycol. The TtGH8 crystals containing xylobiose and xylotriose were both soaked for 30 s in a solution consisting of the well solution, 30% ethylene glycol and 150 m*M* xylobiose/xylotriose. TtGH8 mutant crystals were soaked in 20 m*M* xylohexose (0.1 *M* HEPES pH 6.8, 0.2 *M* ammonium sulfate, 20% PEG 6000) for 15 min to produce a product complex and for 10 s to produce a substrate complex and were dipped in a cryosolution consisting of 30% ethylene glycol before cooling. Crystal data sets were collected on beamlines I02 and I04 at Diamond Light Source.

Data sets were processed using *xia*2/*DIALS* (Winter, 2010 ▸; Winter *et al.*, 2018 ▸), with the outer resolution limits defined by CC_1/2_ > 0.5. Molecular replacement (*Phaser*; McCoy *et al.*, 2007 ▸) and refinement (*REFMAC*; Murshudov *et al.*, 2011 ▸) were carried out using the *CCP*4*i*2 pipeline (Potterton *et al.*, 2018 ▸). The TtGH8 structure was solved by molecular replace­ment using the *CCP*4 implementation of *MOLREP* (Vagin & Teplyakov, 2010 ▸) and default parameters with the protein atoms only of PDB entry 1wu4, the GH8 reducing-end-xylose releasing exo-oligo­xylanase from *Bacillus halodurans* C-125 (Fushinobu *et al.*, 2005 ▸). Manual manipulation in *Coot* (Emsley *et al.*, 2010 ▸) followed by refinement using *REFMAC* was cycled several times to a give a final *R* factor and *R*_free_ of 0.15 and 0.17, respectively. Protein–ligand complex structures were solved by molecular replacement using the *CCP*4 implementation of *MOLREP* (Vagin & Teplyakov, 2010 ▸) with the unliganded TtGH8 enzyme structure as the search model. Ligands were modelled using *JLigand* (Lebedev *et al.*, 2012 ▸). Structural analysis and figure preparation was carried out in *CCP*4*mg* (McNicholas *et al.*, 2011 ▸).

## 3. Results   

### 3.1. Catalytic activity of TtGH8   

*T. turnerae* possesses a wide variety of glycoside hydrolases spanning many different families and potential substrate specificities. Given its possible relevance to xylan degradation and perhaps even marine colonization of wood, DNA encoding the predicted catalytic domain (residues 41–436; Supplementary Table S1) of the GH8 enzyme of the bacterium (TtGH8; locus tag TERTU_4506) was cloned into an *E. coli* expression vector. The gene sequence was codon-optimized by GenScript (DNA sequence given in Supplementary Table S2) and designed with a 3C protease-removable N-terminal hexahistidine tag. *E. coli* containing the recombinant plasmid produced high levels of soluble TtGH8, which was purified using nickel-affinity chromatography and gel-filtration chromatography. The identity of pure TtGH8, as judged by SDS–PAGE, was confirmed by electrospray ionization mass spectrometry (ESI-MS, data not shown).

Initial screening for likely binding ligands/substrates was performed using thermal shift analysis and a panel of oligo/polysaccharides (xyloglucan, rye arabinoxylan, birchwood xylan, shrimp shell chitin, Avicel, cellohexaose, cellobiose, xylobiose and xylohexaose). Significant increases in melting temperature were only observed in conditions containing xylan or xylohexaose, which resulted in a 2.1°C (apo 55.2°C, with xylan 57.3°C) and 2.9°C (apo 57.2°C, with xylohexaose 60.1°C) increase in melting temperature, respectively (Supplementary Fig. S1).

Given the known specificities of family GH8 members (available at http://www.cazy.org/GH8.html), chitosanase (EC 3.2.1.132), cellulase (EC 3.2.1.4), licheninase (EC 3.2.1.73), endo-1,4-β-xylanase (EC 3.2.1.8) and reducing-end xylose-releasing exo-oligoxylanase (EC 3.2.1.156), the increase in melting temperature for TtGH8 suggested activity as a xylan­ase. TtGH8 was therefore incubated (18 h) with a variety of potential substrates and the reaction products were monitored by thin-layer chromatography (Fig. 2 ▸ *a*). TtGH8 was tested against wheat arabinoxylan (WAX), rye arabinoxylan (RAX), corn arabinoxylan (in the aleurone layer coating the seeds known as the bran fraction; CAX), birchwood xylan (BX), mixed-linkage xylan (MLX), xyloglucan (XG) and also β-1,4-linked xylo-oligosaccharides with degrees of polymerization (DP) from 2 to 6. TLC clearly demonstrated that TtGH8 acts as a xylanase, with xylotriose (X3) the predominant product and with activity on WAX, RAX, BX and MLX clearly evident. Xylotetraose (X4) was the minimal length xylooligosaccharide that could act as a substrate for TtGH8 observable by TLC. The TLC results were confirmed using liquid chromatography linked to mass spectrometry (Supplementary Fig. S2). TtGH8 was observed to cleave X6 into X3, X5 into X3 and X2, and X4 into X3 (X1 could not be observed). Analysis of the soluble products removed by centrifugation after incubation of the protein with birchwood xylan (1 mg ml^−1^, 37°C, 18 h) indeed confirmed X3 as the dominant product (Supplementary Fig. S3).

With the knowledge that TtGH8 acts as a xylanase, we next sought to determine the kinetic parameters for the action of TtGH8 both by substrate-depletion analysis using high-performance anion-exchange chromatography with pulsed amperometric detection (HPAEC-PAD) and by reducing-sugar assays with 3,5-dinitrosalicyclic acid (DNSA). HPAEC-PAD analysis (Figs. 2 ▸ *b* and 2 ▸ *c*) yielded a maximal activity, *k*_cat_/*K*_m_, of 7.5 × 10^7^ *M* ^−1^ min^−1^ for X6, with X5 and X4 being considerably worse substrates, with *k*_cat_/*K*_m_ values some five and 123 times lower, respectively (Table 1 ▸). Such values are typical for endo-xylanases, falling as they do between the *k*_cat_/*K*_m_ values reported for GH10 enzymes such as CjXyn10A and CyXyn10C (Pell *et al.*, 2004 ▸). TtGH8 activity against xylose-based polysaccharides was determined using the DNSA reducing-sugar assay (Fig. 3 ▸ *a*). Enzyme activity was monitored over time at five different substrate concentrations, allowing the determination of *k*_cat_/*K*_m_ (Figs. 3 ▸ *b*, 3 ▸ *c* and 3 ▸ *d*). An apparent preference for MLX was observed (1.6 × 10^8^ mg^−1^ ml min^−1^; Table 1 ▸).

### 3.2. Three-dimensional structure of TtGH8   

In order to provide molecular insight into its catalytic properties, as described above, the three-dimensional structure of TtGH8 was determined. TtGH8 was initially crystallized in an apo (unliganded) form and the structure was determined by molecular replacement at a resolution of 1.4 Å (Tables 2 ▸ and 3 ▸), using the protein coordinates only from the structure of the GH8 reducing-end xylose-releasing exo-oligoxylanase from *B. halodurans* C-125 (PDB entry 1wu4; Fushinobu *et al.*, 2005 ▸) as the search model.

**Figure 4:**
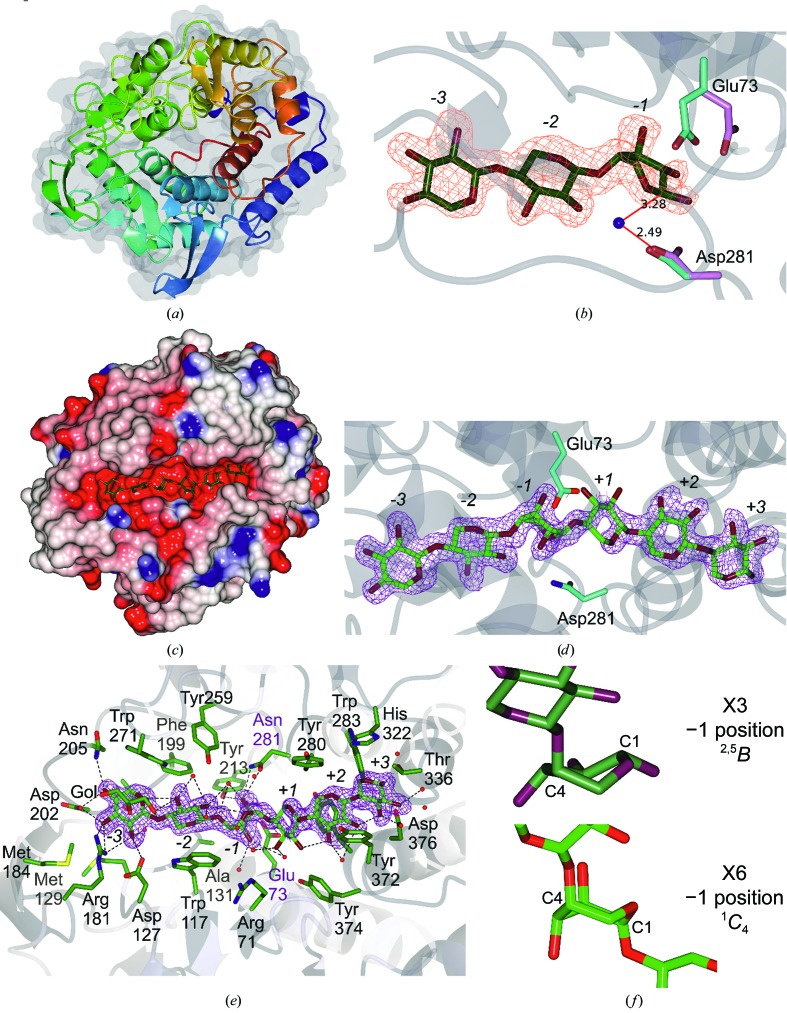
(*a*) Apo TtGH8 ribbon structure (coloured from blue to red) with the overall surface transparent, clearly showing the classical (α/α)_6_ fold. (*b*) TtGH8–xylotriose complex, showing xylotriose bound in sites −3 to −1, with the corresponding maximum-likelihood-weighted *F*_o_ − *F*_c_ ‘difference’ electron density, calculated prior to any incorporation of ligands in refinement, at a contour level of 0.35 e Å^−3^ (approximately 2.5σ). The catalytic residues from the apo form (light blue) and X3 complex (pink) are shown to highlight the position of the scission site and the shift in the catalytic acid (Glu73). The nucleophilic water is shown as a blue sphere, with bonding distances to Asp281 and the catalytic cleavage site (were the substrate longer) depicted. (*c*) TtGH8 D281N–X6 complex shown as a surface (coloured by electrostatic potential), in which xylohexaose can be observed spanning the deep binding cleft. (*d*) TtGH8 D281N–X6 complex shown with xylohexaose spanning the six binding positions −3 to +3 with the associated electron density (contour level 0.35 e^−^ Å^−3^; approximately 2.5σ). The catalytic acid and mutated base are shown in light blue. Here, it can be clearly seen that the position normally taken by the nucleophilic water (sitting below and in line with C1) is unavailable owing to the occupation of O2 caused by a ring flip. (*e*) TtGH8 D281N–X6 complex, showing the nearest neighbours of the X6 ligand (coloured by blending through model); waters are shown in blue, the cryoprotectant ethylene glycol (blue) is shown on the left and hydrogen bonds are depicted by dashed lines. (*f*) Close-up views of the sugar conformations found in the −1 position of the two TtGH8 complexes, showing the conformations, with C1 and C4 labelled, as ^2,5^ *B* and ^1^ *C*_4_ for xylotriose and xylohexaose, respectively. All images were produced using *CCP*4*mg* (McNicholas *et al.*, 2011 ▸).

The first three-dimensional structure of a CAZY family GH8 member was that of *Clostridium thermocellum* CelA (Alzari *et al.*, 1996 ▸). Consistent with the defining family member, TtGH8 exhibits a classical (α/α)_6_ fold with a clear deep substrate-binding groove (Fig. 4 ▸ *a*). *PDBeFold* (Krissinel & Henrick, 2004 ▸) analysis reveals that TtGH8 shares close structural similarity to the reducing-end xylose-releasing exo-oligoxylanase from *B. halodurans* C-125 (Fushinobu *et al.*, 2005 ▸). TtGH8 shares 43% sequence identity, overlaying 344 C^α^ atoms with an r.m.s.d. of 1.1 Å, a *Q* score of 0.7 and a *Z* score of 15.9. The next closest match is the cold-adapted GH8 xylanase from *Pseudoalteromonas haloplanktis* (Collins *et al.*, 2005 ▸) with 35% sequence identity, an r.m.s.d. of 1.4 Å over 341 C^α^ atoms, a *Q* score of 0.6 and a *Z* score of 14.7.

### 3.3. Mechanism of GH8 endoxylanses   

Glycoside hydrolases may act through one of two main mechanisms, leading to retention or inversion of anomeric configuration (reviewed, for example, in Davies & Henrissat, 1995 ▸; Henrissat & Davies, 1997 ▸; Rye & Withers, 2000 ▸). CAZY GH8 enzymes act with inversion of anomeric configuration (Fierobe *et al.*, 1993 ▸) and thus feature two essential catalytic residues: a base to activate the water for nucleophilic attack and an acid to protonate the leaving group for departure. Previous studies on GH8 enzymes have identified a conserved glutamic acid found on helix α2 which functions as the catalytic acid in the inverting mechanism (Alzari *et al.*, 1996 ▸). The equivalent in TtGH8 is Glu73. As in many inverting enzyme families, the location of the base is less clear, and it has been proposed that GH8 may, in fact, be subdivided into groupings based upon the location of the catalytic base (Adachi *et al.*, 2004 ▸). The classical xylanases and endoglucanases (as first reported by Alzari *et al.*, 1996 ▸) are believed to have the base at the end of helix α8, with the equivalent residue in TtGH8 being Asp281. In order to clarify the catalytic residues in TtGH8, crystals were soaked in both xylobiose (X2) and xylotriose (X3) and the resulting structures were refined at 1.4 and 1.8 Å resolution, respectively (Tables 2 ▸ and 3 ▸).

X2 was bound in the −2 and −3 subsites (Supplementary Fig. S4; subsite nomenclature is as discussed by Davies *et al.*, 1997 ▸), hinting at the strength of the −3 subsite, and consistent with the product profiles (Fig. 2 ▸ *a*). X3 was observed bound in the −3 to −1 subsites (Fig. 4 ▸ *b*) and confirms the catalytic residue proposals, with Glu73 (which has rotated from its position in the apo structure into this more catalytically relevant position) interacting with the O1 hydroxyl and with Asp281 interacting with a water molecule ‘below’ C1 in a position mimicking that which would be expected for hydrolysis with inversion of anomeric configuration. Notably, the −1 subsite sugar is not observed in its low-energy ^4^ *C*_1_ chair conformation, but is instead observed distorted into a ^2,5^ *B* conformation (Fig. 4 ▸ *b*), consistent with proposals for the catalytic itinerary of GH8 enzymes discussed below.

Enzymatic glycoside hydrolysis involves the distortion of the reactive, −1 subsite, sugar into a variety of skew-boat and boat conformations, reflecting the requirements of inline attack and the stereoelectronic requirements of an oxo­carbenium-ion-like transition state (for reviews, see Davies & Williams, 2016 ▸; Speciale *et al.*, 2014 ▸; Davies *et al.*, 2003 ▸, 2012 ▸). The GH8 family has been proposed to go through a ^2,5^ *B*-like transition state, notably because of the observation of sugars with ^2^ *S*_O_ and ^2,5^ *B* conformations in complexes of the CelA endoglucanase (Guérin *et al.*, 2002 ▸) that were subsequently analysed by QM/MM metadynamics (Petersen *et al.*, 2009 ▸).

The previous X3 complex, that of the *P. haloplanktis* cold-adapted xylanase (Collins *et al.*, 2005 ▸; De Vos *et al.*, 2006 ▸), revealed binding in the +1 to +3 subsites, which together with the −3 to −1 observations here approximately defined binding through the six subsites of the enzyme, which is consistent with the inactive-variant (Asp144Ala) complex with X5 observed for the *P. haloplanktis* enzyme by De Vos *et al.* (2006 ▸). In order to observe the hexasaccharide complex of TtGH8 we first made a catalytic variant at the proposed base, TtGH8 D281N; however, this appeared to retain around 0.1% of the catalytic activity (*k*_cat_/*K*_m_ of 1.8 × 10^4^ mg^−1^ ml min^−1^; Table 1 ▸) in the reducing-sugar assay with MLX as substrate, which is consistent with similar mutants in other GH8 systems (Collins *et al.*, 2005 ▸; De Vos *et al.*, 2006 ▸). In addition, structures with X6, with long soak times, all revealed xylotriose product complexes (not shown). A complex with unhydrolysed X6 was therefore obtained by rapid soaking/cooling with a total time of approximately 10 s. Thus, a TtGH8 D281N structure in complex with X6 was obtained at 1.6 Å resolution, revealing X6 bound across the entire substrate-binding groove from subsites −3 to +3 (Figs. 4 ▸ *c*, 4 ▸ *d* and 4 ▸ *e*; Tables 2 ▸ and 3 ▸).

The complex of TtGH8 with xylotriose showed distortion of the −1 xylose into a boat configuration, consistent with past work by others on the conformational itinerary in this family. To our surprise, the TtGH8 D281N–X6 complex revealed something different; namely, the −1 subsite sugar in a completely ring-flipped, southern hemisphere ^1^ *C*_4_ chair conformation (Fig. 4 ▸ *f*). Although this allowed access to a hexasaccharide complex structure, the ring-flipped −1 sugar is unlikely to be representative of a catalytically relevant conformation since its position neither allows protonation of the leaving group by Glu73 nor is there a potential reactive water. Indeed, in the ^1^ *C*_4_ chair conformation the now axial (and ‘down’) O2 occupies the position that should instead be occupied by the nucleophilic water. Indeed, the only other structure in this family in which a substrate spanning the −1 subsite has been observed in anything other than the boat conformation was in the X5 complex of the *P. haloplanktis* GH8 (PDB entry 2b4f; De Vos *et al.*, 2006 ▸). Here, the −1 xylose was undistorted ^4^ *C*_1_ (although with scant density) and featured a position of the catalytic acid (akin to the TtGH8 apo structure) that was not commensurate with its role as a proton donor. This work, together with our current study, therefore highlights the difficulty in analysing substrate-complex structures with xylose-containing oligosaccharides which may be influenced by the integral conformational flexibility of xylose. Importantly, whilst the Asp281Asn variant has allowed definition of the interactions of the −3 to −2 and +1 to +3 subsites well, it highlights the occasional dangers of using inactive variants to study substrate distortion in the −1 site.

## 4. Discussion   

As with recent studies on xylan utilization by the human microbiota (Rogowski *et al.*, 2015 ▸), the digestive system of bivalve molluscs such as marine wood-boring shipworms has the potential to provide a wealth of carbohydrate-active enzymes with potentially beneficial applications. Here, we have studied a potential GH8 xylanase from the shipworm symbiont *T. turnerae*, the genome sequence of which (Yang *et al.*, 2009 ▸) unveiled a treasure chest of carbohydrate-active enzymes. We have shown that TtGH8 is a single-domain endo-xylanase with six catalytically relevant subsites that hydrolyses X6 to yield predominantly xylotriose. The enzyme is active on diverse classical β-1,4-xylans, likely reflecting its role in the host digestion of woody biomass after the possible translocation of bacterial proteins from the gills to the connected gastrointestinal tract of its Teredinidae shipworm host (O’Connor *et al.*, 2014 ▸). The enzyme thus bears similarities to the well studied *P. haloplanktis* ‘cold-adapted’ xylanase, with which TtGH8 shares 33% identity. Intriguingly, TtGH8 shows the highest activity on mixed-linkage β-1,3,β-1,4 xylans, which may reflect a genuine biological adaption to, or at least an accommodation of, these marine substrates. The mixed-linkage marine xylan used in this study is found as a component of the red alga *Palmaria palmata*, and is a polysaccharide that is involved in mechanical support, development and defence (Viana *et al.*, 2011 ▸). Analysis of this polysaccharide suggests a 1:3 ratio of β-1,3:β-1,4 moieties. Whilst pure β-1,4 bonding of xylose residues would result in a threefold screw-axis helical structure, the irregular distribution of β-1,3 sections between variable lengths of β-1,4 sections may cause disruption of this regular conformation (Viana *et al.*, 2011 ▸). Optical rotation alignment further suggests that mixed-linkage xylan exhibits a ‘random-coil’ structure; unlike linear β-1,4-xylan, which may form interactions with other chains, the presence of β-1,3 linkages introduces flexibility which may assist in the solubility (Viana *et al.*, 2011 ▸; Cerezo *et al.*, 1971 ▸). Flexibility may improve the fitting of the polysaccharide into the V-shaped binding site of TtGH8. Improvement in solu­bility owing to the flexible nature of the xylan chain may be a factor in the increased degradation rate exhibited by TtGH8. We would argue, therefore, that the increased activity on MLX may not necessarily reflect the requirement for a β-1,3-linked xylose at one or more of the subsites, but confers increased solubility and thus enzyme access. It is also possible, given its marine environment, that the specificity of the enzyme has adapted to potential terrestrial xylans (β-1,4-xylans) and marine xylans (MLXs). It should be noted, however, that only *k*_cat_/*K*_m_ values were determined and not the individual kinetic constants. This was because the maximum soluble substrate concentration was much lower than *K*_m_, as indicated by the linear relationship between the rate and the substrate concentration. It is possible, therefore, that the difference in activity reflects a variation in *K*_m_ values that may not reflect the binding affinities but the actual concentrations of available substrate in two very different xylans. MLX has not been extensively used as a substrate in the analysis of xylanase activity. Exploring whether MLXs feature as the optimum substrate for all xylanases, or only those enzymes exposed to a marine system, will provide insight into the environmental selection pressures that influence glycoside hydrolase activity. In a discussion of shipworm larvae, Turner states that whilst shipworm larvae are quick to settle into burrows after extrusion from the adult, most wooden structures are covered in a ‘protective forest’ of various organisms, including algae, in which the young shipworm larvae may swim before settlement (Turner, 1966 ▸). Bacterial symbionts are passed onto shipworm young, so it is possible that algal particles are digested using enzymes such as TtGH8 before or during larvae settlement. Such analyses highlight how shipworms and their symbionts offer a plethora of possibilities for novel enzyme discovery and application for biotechnology and biofuels.

## Supplementary Material

PDB reference: GH8 xylanase from *Teredinibacter turnerae*, 6g00

PDB reference: complex with xylobiose, 6g09

PDB reference: complex with xylotriose, 6g0b

PDB reference: catalytic mutant, complex with xylohexaose, 6g0n

Supplementary Tables and Figures.. DOI: 10.1107/S2059798318009737/cb5108sup1.pdf

## Figures and Tables

**Table 1:** Catalytic activities of TtGH8 on xylan substrates as determined by HPAEC-PAD (oligosaccharides) or DNSA reducing-sugar assay (polysaccharides)

Substrate	*k*_cat_/*K*_m_ †
Oligosaccharides	
Xylohexaose	7.5 × 10^7^ ± 1.1 × 10^6^
Xylopentaose	1.4 × 10^7^ ± 1.4 × 10^6^
Xylotetraose	6.1 × 10^5^ ± 3.1 × 10^4^
Polysaccharides	
BX	1.8 × 10^7^ ± 4 × 10^6^
WAX	6.3 × 10^6^ ± 5 × 10^5^
MLX (wild type)	1.6 × 10^8^ ± 4 × 10^6^
MLX (Asp281Asn)	1.8 × 10^4^ ± 1 × 10^3^

† Values for oligosaccharides are given in *M* ^−1^ min^−1^ and those for polysaccharides are given in mg^−1^ ml min^−1^.

**Table 2:** Data-collection and processing statistics for TtGH8 and the TtGH8 mutant with and without ligands, and the TtGH8 mutant–X6 complex Values in parentheses are for the outer shell.

	Native TtGH8	TtGH8–X2	TtGH8–X3	TtGH8 mutant–X6
Diffraction source	Diamond Light Source	Diamond Light Source	Diamond Light Source	Diamond Light Source
Wavelength (Å)	0.98	0.98	0.98	0.98
Temperature (K)	100	100	100	100
Space group	*P*2_1_2_1_2_1_	*P*2_1_2_1_2_1_	*P*2_1_2_1_2_1_	*P*2_1_2_1_2_1_
*a*, *b*, *c* (Å)	61.5, 73.0, 90.9	61.7, 79.0, 87.6	59.7, 80.5, 88.0	62.0, 79.7, 88.0
α, β, γ (°)	90.0, 90.0, 90.0	90.0, 90.0, 90.0	90.0, 90.0, 90.0	90.0, 90.0, 90.0
Resolution range (Å)	51.0–1.40 (1.42–14.0)	50.4–1.40 (1.42–1.40)	59.4–1.80 (1.84–1.80)	60.97–1.80 (1.84–1.80)
Total No. of reflections	342816	530153	316157	313138
No. of unique reflections	80610	84123	40092	40046
Completeness (%)	99.4 (99.9)	99.1 (89.6)	100.0 (100.0)	99.0 (98.0)
Multiplicity	4.3 (4.3)	6.3 (4.1)	7.9 (7.7)	7.8 (8.0)
〈*I*/σ(*I*)〉	9.6 (1.7)	10.1 (1.3)	8.5 (1.6)	8.2 (2.0)
CC_1/2_	0.997 (0.670)	0.997 (0.503)	0.994 (0.528)	0.992 (0.639)
*R*_p.i.m._	0.045 (0.414)	0.051 (0.549)	0.080 (0.611)	0.109 (0.970)
Overall *B* factor from Wilson plot (Å^2^)	9	9	13	12

**Table 3:** Structure solution and refinement of TtGH8 and the TtGH8 mutant with and without ligands, and the TtGH8 mutant–X6 complex Values in parentheses for the outer shell.

	Native TtGH8	TtGH8–X2	TtGH8–X3	TtGH8 mutant–X6
Resolution range (Å)	51.0–1.40 (1.42–1.40)	50.4–1.40 (1.42–1.40)	59.4–1.80 (1.84–1.80)	60.97–1.80 (1.84–1.80)
Completeness (%)	99.4 (99.9)	99.1 (89.6)	100.0 (100.0)	99.0 (98.0)
No. of reflections, working set	80540	84054	40032	39993
No. of reflections, test set	3924	4274	1868	1942
Final *R*_cryst_	0.15	0.16	0.17	0.17
Final *R*_free_	0.17	0.18	0.20	0.20
Cruickshank DPI	0.052	0.051	0.111	0.116
No. of non-H atoms				
Protein	3163	3145	3125	3136
Ion	1	—	—	—
Ligand	25	23	28	61
Water	310	330	164	194
R.m.s. deviations				
Bonds (Å)	0.017	0.016	0.012	0.012
Angles (°)	1.70	1.68	1.51	1.50
Average *B* factors (Å^2^)				
Protein	14	12	17	16
Ion	18	—	—	—
Ligand	28	15	20	26
Water	27	22	24	22
Ramachandran plot				
Most favoured (%)	98.2	97.5	98.0	97.5
Allowed (%)	1.8	2.5	2.0	2.5
PDB code	6g00	6g09	6g0b	6g0n
