# Supplementary material for: Structure and function of a glycoside hydrolase family 8 endoxylanase from Teredinibacter turnerae
Source: Acta Crystallogr D Struct Biol. 2018 Oct 2;74(Pt 10):946–55. doi: 10.1107/S2059798318009737 (PMC6173055; doi:10.1107/S2059798318009737)
Supplement: Supplementary file 1 [file d-74-00946-sup1.pdf]

STRUCTURAL
BIOLOGY

Volume 74 (2018)

Supporting information for article:

Structure and function of a GH8 endoxylanase from *Teredinibacter turnerae*

Claire A. Fowler, Glyn R. Hemsworth, Fiona Cuskin, Sam Hart, Johan Turkenburg, Harry Gilbert, Paul H. Walton and Gideon J. Davies

Table S1 TtGH8 (CAZy reference TERTU_4506) DNA catalytic domain sequence involving residues 41-436.

GCTGGTGCCGTTGCTACCGGCGAGTACCGCAATCTGTTTGCCGAAATCGGAAAAAGCGAAATAGACATCC<br>AGCGCAAAATTGACGAGGCGTTTCAGCACTTGTTTTATGGCGACGCGAAAGATGCAGCTGTCTACTATCA<br>AGCGGGTGGAAACGAGAATGGTCCACTCGCATATGTTTACGATGTGAACAGCAATGACGTGCGCTCAGA<br>AGGCATGAGCTACGGCATGATGATTACTGTTCAAATGGACAAAAAAGCCGAGTTCGATGCAATCTGGAA<br>CTGGGCGAAAACCTATATGTATCAAGACTCCCCCACGCATCCAGCGTTTGGTTACTTTGCCTGGTCCATGC<br>GCCGCGATGGTGTCGCCAATGACGATATGCCAGCGCCAGATGGCGAGGAATATTTCGTGACCGCTCTCTA<br>TTTCGCCGCCGCCCGCTGGGGTAATGGCGAAGGTATTTTCAACTACCAACAGGAAGCGGACACCATTTTG<br>AGCCGCATGCGCCACCGCCAGGTGATCACCGGCCCAACCAATCGCGGAGTAATGACTGCGACCAATCTG<br>TTCCACCCGGAAGAGGCGCAAGTGCGCTTCACGCCCGACATCAATAATGCTGATCATACAGACGCGTCTT<br>ACCATCTGCCCTCGTTCTATGAAATTTGGGCACGTGTCGCGCCGCAAGAAGATCGCGCGTTTTGGGCCAA<br>AGCGGCCGATGTGAGCCGCGACTATTTTGCCAAAGCCGCCCACCCTGTCACTGCGTTAACACCGGACTAC<br>GGTAATTTTGATGGCACCCCGTGGGCGGCATCCTGGCGGCCGGAGTCGGTAGATTTTCGATACGATGCCT<br>GGCGTTCCGTCATGAACTGGTCCATGGACTATGCCTGGTGGGGCAAAGATTCAGGCGCACCCGCGCGCA<br>GTGATAAATTACTCGCGTTCTTCGAAACCCAGGAAGGCAAAATGAACCACCTCTATAGCCTGGATGGCAA<br>ACCGCTGGGTGGTGGACCGACCCTCGGCCTAATTTCCATGAATGCAACGGCAGCTATGGCAGCTACTGAT<br>CCCCGCTGGCACAATTTTGTGGAAAAGCTCTGGCAACAACAACCCCCCACAGGGCAATACCGGTACTAC<br>GACGGTGTTCTATACCTGATGGCGCTGCTACATTGCGCTGGGGAGTACAAAGCGTGGATCCCCGACGGGG<br>AATAA

Table S2 TtGH8 macromolecule production information. The TtGH8 catalytic domain was identified and a construct produced without the associated host periplasmic leader sequence and linker section. A hexahistidine tag and 3C protease cleavage site was built into the construct (flanked by Ncol and Xhol restriction sites) produced by GenScript, which was codon optimised for use in E.coli.

Source organism	*Teredinibacter turnerae*
DNA source	Optimised sequence obtained from GenScript
Forward primer	Ncol
Reverse primer	Xhol
Cloning vector	One Shot Top10
Expression vector	pET-28a
Expression host	*E.coli* BL21 (DE3)
Codon Optimised DNA Sequence	ATGGGCAGCAGCCATCATCATCATCATCACAGCAGCGG<br>CCTGGAAGTTCTGTTCCAGGGACCAGCAGCGGGTGCGG<br>TGGCGACCGGTGAATACCGTAACCTGTTCGCGGAGATTG<br>GCAAGAGCGAAATCGACATTCAACGTAAAATCGATGAA<br>GCGTTCCAGCACCTGTTTTATGGCGACGCGAAGGATGCG<br>GCGGTTTACTATCAAGCGGGTGGCAACGAGAACGGTCC<br>GCTGGCGTACGTGTATGACGTTAACAGCAACGATGTGC<br>GTAGCGAGGGTATGAGCTACGGCATGATGATTACCGTTC<br>AAATGGACAAGAAAGCGGAATTTGATGCGATCTGGAAC<br>TGGGCGAAAACCTACATGTATCAAGACAGCCCGACCCA<br>CCCGGCGTTCGGTTATTTTGCGTGGAGCATGCGTCGTGA

	TGGTGTGGCGAACGATGATATGCCGGCGCCGGATGGCG<br>AGGAATACTTCGTTACCGCGCTGTATTTTGCGGCGGCGC<br>GTTGGGGTAACGGCGAGGGTATCTTCAACTACCAGCAA<br>GAAGCGGATACCATTCTGAGCCGTATGCGTCACCGTCAA<br>GTGATCACCGGTCCGACCAACCGTGGCGTTATGACCGCG<br>ACCAACCTGTTCCACCCGGAGGAAGCGCAAGTGCGTTTT<br>ACCCCGGACATTAACAACGCGGACCACACCGATGCGAG<br>CTACCACCTGCCGAGCTTTTATGAGATCTGGGCGCGTGT<br>TGCGCCGCAGGAAGATCGTGCGTTTTGGGCGAAGGCGG<br>CGGATGTGAGCCGTGATTACTTTGCGAAGGCGGCGCAC<br>CCGTTACCGCGCTGACCCCGGACTATGGTAACTTCGATG<br>GTACCCCGTGGGCGGCGAGCTGGCGTCCGGAGAGCGTG<br>GACTTTCGTTACGATGCGTGGCGTAGCGTTATGAACTGG<br>AGCATGGACTATGCGTGGTGGGGTAAAGATAGCGGTGC<br>GCCGGCGCGTAGCGACAAACTGCTGGCGTTCTTTGAGAC<br>CCAAGAAGGTAAAATGAACCACCTGTACAGCCTGGACG<br>GTAAGCCGCTGGGTGGCGGTCCGACCCTGGGTCTGATTA<br>GCATGAACGCGACCGCGGCGATGGCGGCGACCGACCCG<br>CGTTGGCACAACTTCGTGGAAAAGCTGTGGCAGCAACA<br>GCCGCCGACCGGCCAGTACCGTTACTATGACGGCGTTCT<br>GTATCTGATGGCGCTGCTGCACTGCGCGGGCGAGTACA<br>AAGCGTGGATCCCGGATGGCGAATAACTCGAG
Complete amino acid sequence of the construct produced	M G S S H H H H H H S S G L E V L F Q G P A A G A V A T G<br>E Y R N L F A E I G K S E I D I Q R K I D E A F Q H L F Y G<br>D A K D A A V Y Y Q A G G N E N G P L A Y V Y D V N S N<br>D V R S E G M S Y G M M I T V Q M D K K A E F D A I W N<br>W A K T Y M Y Q D S P T H P A F G Y F A W S M R R D G V<br>A N D D M P A P D G E E Y F V T A L Y F A A A R W G N G<br>E G I F N Y Q Q E A D T I L S R M R H R Q V I T G P T N R G<br>V M T A T N L F H P E E A Q V R F T P D I N N A D H T D A<br>S Y H L P S F Y E I W A R V A P Q E D R A F W A K A A D V<br>S R D Y F A K A A H P V T A L T P D Y G N F D G T P W A A<br>S W R P E S V D F R Y D A W R S V M N W S M D Y A W W<br>G K D S G A P A R S D K L L A F F E T Q E G K M N H L Y S<br>L D G K P L G G G P T L G L I S M N A T A A M A A T D P R<br>W H N F V E K L W Q Q Q P P T G Q Y R Y Y D G V L Y L M<br>A L L H C A G E Y K A W I P D G E Stop L E

Table S3 TtGH8 mutant macromolecule production information. Primers for point mutation were designed for the TtGH8 construct and implemented using NEB Q5 site mutagenesis kit.

Source organism	*Teredinibacter turnerae*
DNA source	TtGH8 Optimised sequence obtained from GenScript
Forward primer	CTTTCGTTACAACGCGTG GCGTAGCG (Asp 281- Asn)
Reverse primer	TCCACGCTCTCCGGACG C (Asp 281- Asn)
Cloning vector	One Shot Top10
Expression vector	pET-28a
Expression host	*E.coli* BL21 (DE3)
Complete amino acid sequence of the construct produced	M G S S H H H H H H S S G L E V L F Q G P A A G A V A T G E Y R N L F A E I G K S E I D I Q R K I D E A F Q H L F Y G D A K D A A V Y Y Q A G G N E N G P L A Y V Y D V N S N D V R S E G M S Y G M M I T V Q M D K K A E F D A I W N W A K T Y M Y Q D S P T H P A F G Y F A W S M R R D G V A N D D M P A P D G E E Y F V T A L Y F A A A R W G N G E G I F N Y Q Q E A D T I L S R M R H R Q V I T G P T N R G V M T A T N L F H P E E A Q V R F T P D I N N A D H T D A S Y H L P S F Y E I W A R V A P Q E D R A F W A K A A D V S R D Y F A K A A H P V T A L T P D Y G N F D G T P W A A S W R P E S V D F R Y N A W R S V M N W S M D Y A W W G K D S G A P A R S D K L L A F F E T Q E G K M N H L Y S L D G K P L G G G P T L G L I S M N A T A A M A A T D P R W H N F V E K L W Q Q Q P P T G Q Y R Y Y D G V L Y L M A L L H C A G E Y K A W I P D G E Stop L E

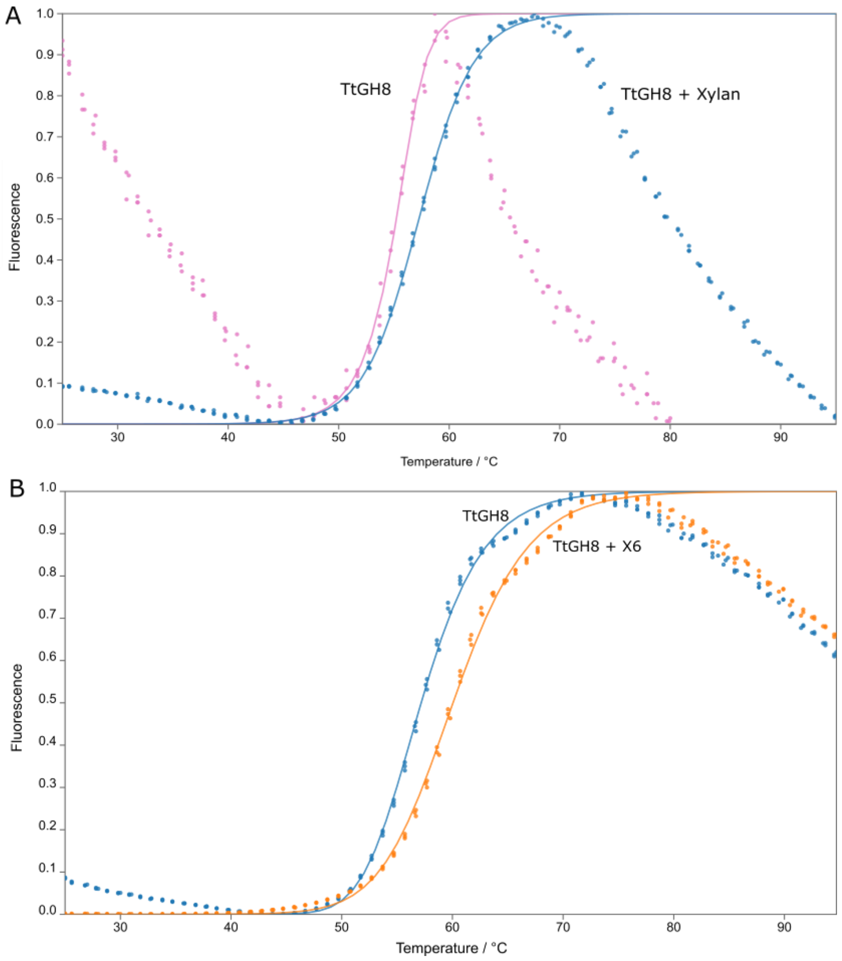

Figure S1 Thermal shift analysis of apo TtGH8 (22 µM) and incubation with A) birchwood xylan (1 mg/ml) and B) xylohexose (X6, 10 mM) in the presence of SYPRO orange. Fluorescence (shown as normalised values) indicates interaction of the SYPRO orange dye with aromatic residues commonly found within a protein structure. Increasing fluorescence as temperature increases models the denaturation of the protein. Analysis of the protein melting curves was completed using the JTSA online tool developed by Paul Bond, available at URL http://paulsbond.co.uk/jtsa/#/input. Addition of xylan and xylohexaose (X6) shifted the melting temperature of the apo protein from 55.2 -57.3 °C and 57.2 - 60.1 °C respectively. In general, a significant shift in temperature is deemed to be at least 1 °C and would indicate an interaction of the protein with the substrate. A positive shift in melting temperature of 2.1 °C due to xylan incubation and 2.9 °C with xylohexaose is indicative of TtGH8 activity/binding.

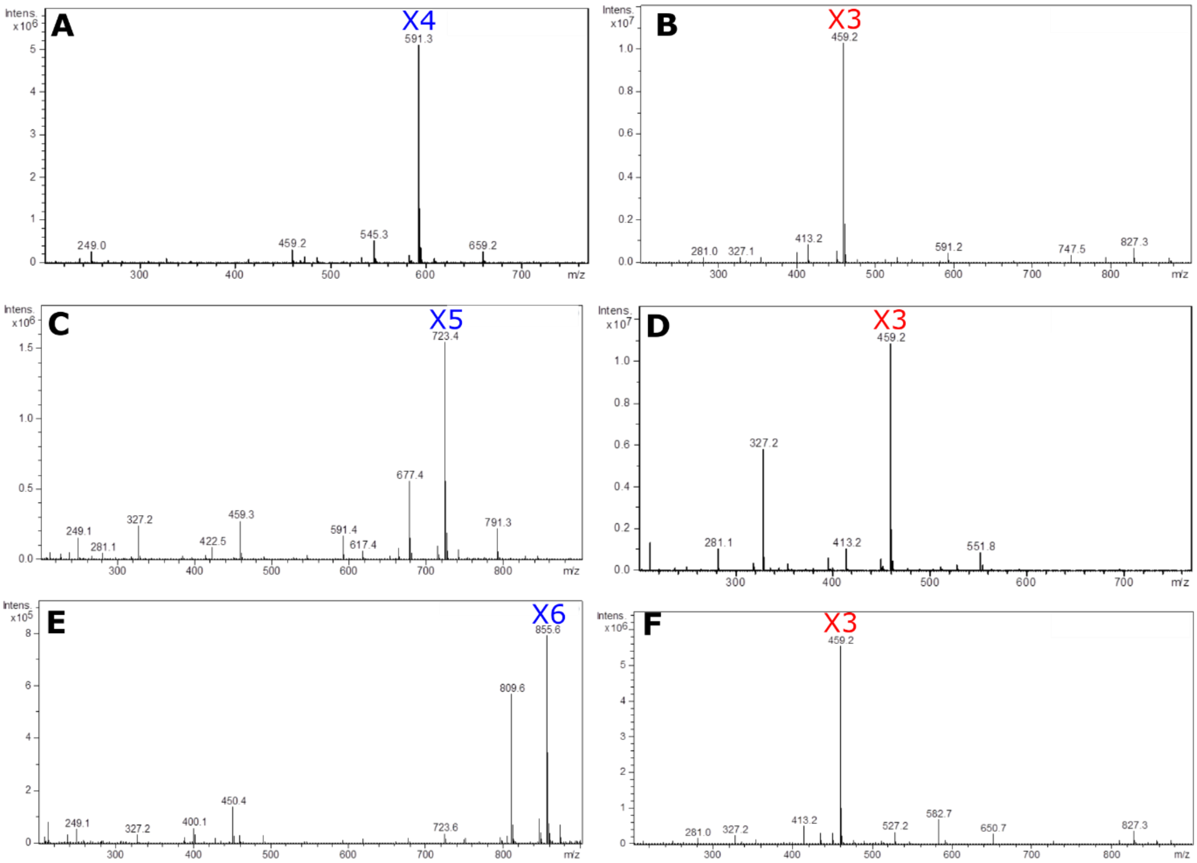

Figure S2 LCMS results of TtGH8 against larger xylooligosaccharides. A) Xylotetraose (591.3 m/z, formic acid adduct) B) Reaction products of TtGH8 and xylotetraose, producing xylotriose (459.2 m/z formic acid adduct). Xylose is not seen due to detections limits of the equipment. C) Xylopentaose (723.4 m/z formic acid adduct) D) Reaction products of TtGH8 and xylopentaose, producing xylotriose (459.2 m/z formic acid adduct) and xylobiose (327.2 m/z formic acid adduct). E) Xylohexaose (855.6 m/z formic acid adduct) F) Reaction products of TtGH8 and xylohexaose, producing only xylotriose (459.2 m/z formic acid adduct).

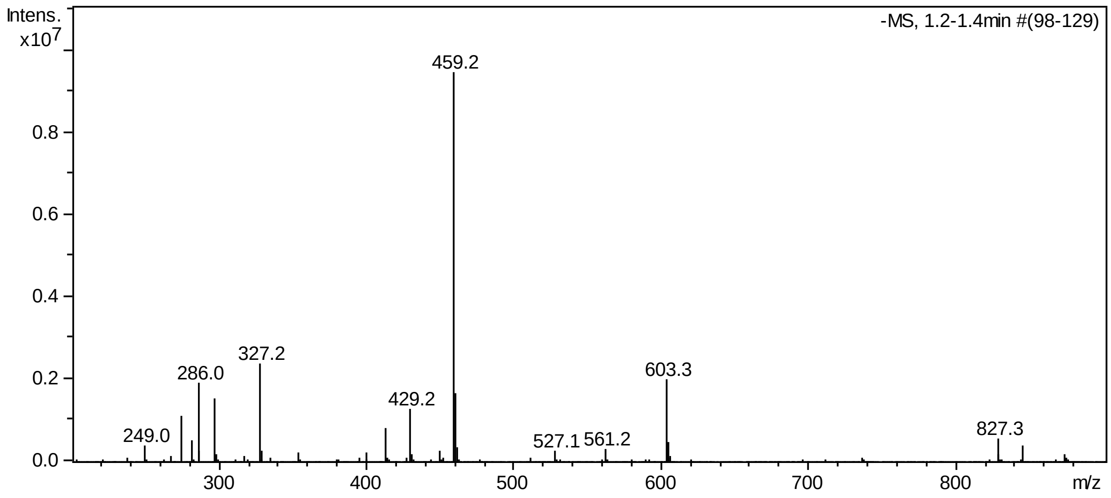

Figure S3 Liquid chromatography mass spectrum (LCMS-Dionex system) of soluble reaction products (run on a Cosmosil Sugar-D HPLC column) generated after incubation of TtGH8 (1 μM) with birchwood xylan (1 mg/ml) for 18 hr at 37 °C. Xylotriose and xylobiose are observed as formic acid adducts at 327.2 and 459.2 m/z respectively.

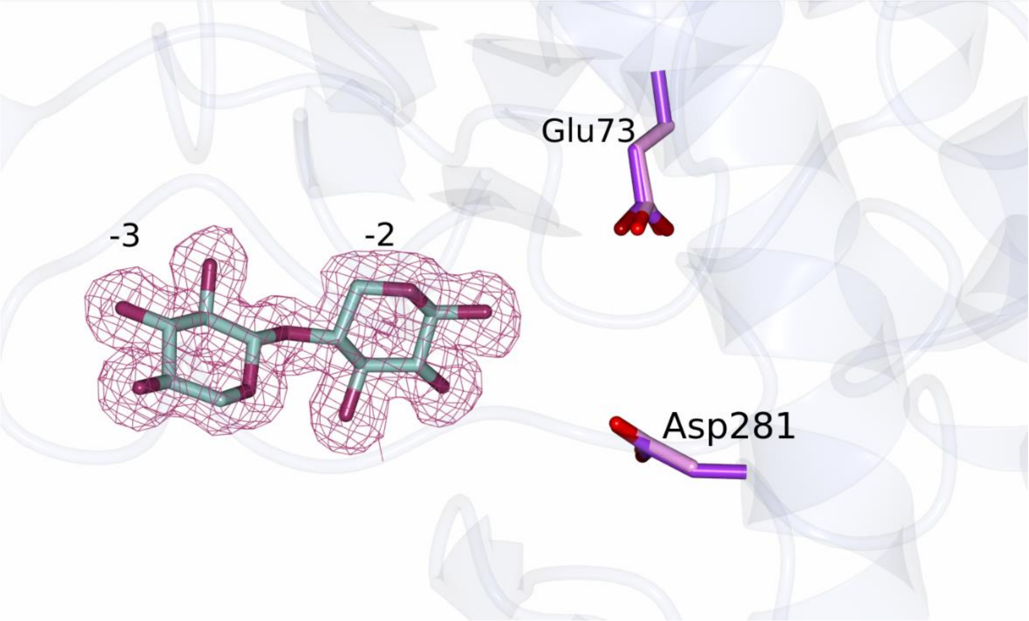

Figure S4 TtGH8 xylobiose complex, showing xylobiose bound in sites -3 to -2, with the corresponding maximum likelihood weighted Fo-Fc "difference" electron density, calculated *prior* to any incorporation of ligands in refinement, at contour level of 0.35 electrons / $Å^3$ (approx. 2.5σ). The catalytic residues in the xylobiose complex and the unliganded enzyme are shown in purple and light-pink, respectively.
